# Entropic Imaging of Cataract Lens: An *In Vitro* Study

**DOI:** 10.1371/journal.pone.0096195

**Published:** 2014-04-23

**Authors:** Zhuhuang Zhou, Chih-Chung Huang, K. Kirk Shung, Po-Hsiang Tsui, Jui Fang, Hsiang-Yang Ma, Shuicai Wu, Chung-Chih Lin

**Affiliations:** 1 Biomedical Engineering Center, College of Life Science and Bioengineering, Beijing University of Technology, Beijing, China; 2 Department of Biomedical Engineering, National Cheng Kung University, Tainan, Taiwan; 3 NIH Resource on Medical Ultrasonic Transducer Technology, Department of Biomedical Engineering, University of Southern California, Los Angeles, California, United States of America; 4 Department of Medical Imaging and Radiological Sciences, College of Medicine, Chang Gung University, Taoyuan, Taiwan; 5 Institute for Radiological Research, Chang Gung University and Chang Gung Memorial Hospital, Taoyuan, Taiwan; 6 Ph.D. Program in Biomedical Engineering, Chang Gung University, Taoyuan, Taiwan; 7 Graduate Institute of Clinical Medical Sciences, College of Medicine, Chang Gung University, Taoyuan, Taiwan; 8 Department of Computer Science and Information Engineering, Chang Gung University, Taoyuan, Taiwan; University of Arkansas for Medical Sciences, United States of America

## Abstract

Phacoemulsification is a common surgical method for treating advanced cataracts. Determining the optimal phacoemulsification energy depends on the hardness of the lens involved. Previous studies have shown that it is possible to evaluate lens hardness via ultrasound parametric imaging based on statistical models that require data to follow a specific distribution. To make the method more system-adaptive, nonmodel-based imaging approach may be necessary in the visualization of lens hardness. This study investigated the feasibility of applying an information theory derived parameter – Shannon entropy from ultrasound backscatter to quantify lens hardness. To determine the physical significance of entropy, we performed computer simulations to investigate the relationship between the signal-to-noise ratio (SNR) based on the Rayleigh distribution and Shannon entropy. Young's modulus was measured in porcine lenses, in which cataracts had been artificially induced by the immersion in formalin solution in vitro. A 35-MHz ultrasound transducer was used to scan the cataract lenses for entropy imaging. The results showed that the entropy is 4.8 when the backscatter data form a Rayleigh distribution corresponding to an SNR of 1.91. The Young's modulus of the lens increased from approximately 8 to 100 kPa when we increased the immersion time from 40 to 160 min (correlation coefficient *r* = 0.99). Furthermore, the results indicated that entropy imaging seemed to facilitate visualizing different degrees of lens hardening. The mean entropy value increased from 2.7 to 4.0 as the Young's modulus increased from 8 to 100 kPa (*r* = 0.85), suggesting that entropy imaging may have greater potential than that of conventional statistical parametric imaging in determining the optimal energy to apply during phacoemulsification.

## Introduction

A cataract is a clouding of the normally transparent crystalline lens of the eye, resulted from factors such as aging, lens damage, and heredity. The formation of a cataract can be considered a process of lens fibrosis, whereby both protein aggregation and fiber coemption occur inside the lens causing the lens hardness to increase [1], [2]. The initial stage of cataract formation can generally be mitigated by applying a medical eyewash. However, advanced cataracts require treatment with phacoemulsification to replace the turbid lens with an artificial one, thereby restoring clear vision. Lens hardness exerts a marked effect on the optimal ultrasonic energy in phacoemulsification, in which optimizing the energy improves the efficiency of the surgery and reduces the injury to the lens capsule and corneal endothelium. Therefore, evaluating the lens hardness is critical to facilitate successful phacoemulsification surgery.

Ultrasound, an acoustic wave with a frequency exceeding 20 kHz, has been widely used for imaging the eye in ophthalmic applications. In addition to morphology analysis, ultrasound can be applied to evaluate the hardness of a lens. Previous studies have demonstrated that acoustic attenuation and the speed of sound tend to increase as the hardness of a cataract lens increases [1], [3]–[5]. The major shortcoming of applying quantitative ultrasound parameters is that the local variation in lens hardness is difficult to estimate. Employing high-frequency ultrasound B-mode imaging to image a cataract lens is a feasible method for addressing this problem. In principle, the echo intensity of a cataract lens differs from that of a normal lens; specifically, optical opacities produce acoustic inhomogeneity inducing high echogenicity [6]. However, relying on echo intensity alone as a criterion for grading and staging of lens hardening has been shown to be problematic because of the poor reproducibility. Our previous studies, which investigated cataracts of porcine eyes in vitro with high-frequency ultrasound, showed that the B-mode image revealed only the structures of the cataract lens, and it was difficult to assess lens hardness merely from the echo intensity [7], [8]. In addition, compared with the conventional B-mode imaging, which shows the backscatter intensity, ultrasound parametric imaging capable of providing additional information associated with scatterer arrangements [9] based on the statistical distribution of the backscatter envelope was found to perform satisfactorily in characterizing the degree of lens hardening [7]. Several studies have shown that high-frequency statistical parametrical imaging could distinguish both global and local variations in lens hardness [7], [8].

Although the hardness of the cataract lens can be characterized by applying statistical distributions to model the envelope statistics, one constraint in employing a statistical model is that the backscatter data must be assumed to follow a specific distribution [10]. This requirement may not always be satisfied because adjusting the settings in an ultrasound system can alter the statistical distribution of raw data, rendering these statistical models inapplicable. To address this limitation and ensure that the method is more system-adaptive, developing a nonmodel-based imaging approach for visualizing lens hardness is preferred.

Among all possibilities, an information theory derived parameter, entropy, is a suitable candidate for developing a system-adaptive imaging method based on analyzing backscatter statistics. Entropy is a measure of uncertainty in a random variable. An increase in entropy indicates a change in the properties of backscatter signals from regular, random, to complex, corresponding to an increase in signal uncertainty. Entropy is not a model-based measure but rather is a function of the variable's probability density function (PDF); therefore, it may reflect the physical significance associated with the backscatter statistics to a degree. This also explains why the entropy 1) increases as the density of scatterers in the resolution cell increases, 2) increases as the scatterer spacing decreases, and 3) is independent of scattering amplitude [11]. Moreover, this parameter does not require the backscatter data to follow a specific distribution, providing a chance to construct methods that are more flexible in practical applications.

Shannon entropy is the most frequently used entropy in information theory. Therefore, this study examined the feasibility of applying the Shannon entropy of ultrasound backscatter data to image the cataract lens. The Shannon entropy *H*(*X*) of a continuous random variable *X* with the PDF *f*(*x*) is defined in [12] and is expressed as follows:
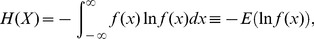
(1)where *E*(.) is the expected value operator. Initially, we investigated the relationship between the signal-to-noise ratio (SNR) and Shannon entropy by conducting computer simulations to better understand the physical meaning of Shannon entropy. In particular, we determined the Shannon entropy threshold for classifying regular (a partially developed speckle), random (a fully developed speckle), and complex (a fully developed speckle with clustered scatterers). Next, we measured the Young's modulus in cataracts at different stages that were artificially induced in porcine lenses in vitro. The samples were scanned with a high-frequency ultrasound transducer to obtain the B-mode and entropy images, and the entropy parameters in the regions of interest (ROI) were compared with the Young's modulus to evaluate the performance of the entropy image in quantifying the lens hardness.

## Materials and Methods

### Computer Simulations

**Figure 1:**
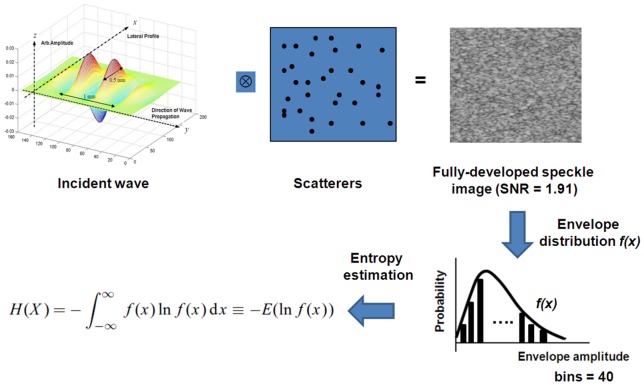
Simulation procedure for determining the threshold of Shannon entropy to classify regular (partially developed speckle), random (fully developed speckle), and complex (fully developed speckle with clustered scatterers).

To explore the physical meaning of Shannon entropy, the entropy value under the condition of a fully developed speckle image was estimated, according to the simulation procedure in Figure 1. A system-based model was used to simulate ultrasonic signals. The backscatter radiofrequency (RF) signals can be modeled as [13].
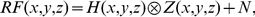
(2)where *x* and *z* respectively denote the axes along the lateral and elevation dimensions, *y* is the axial direction along which the incident ultrasonic wave propagates, *H* represents the transducer transfer function, *Z* is the spatial distribution function of the scatterers, and *N* is signal-independent white noise.

The simulated sampling rate was set at 50 MHz, and the speed of sound was 1540 m/s. To simplify computational complexity, the elevation direction was not considered, and only 2D simulations were performed. First, a homogeneous computer phantom was constructed. A computer phantom is a 2D matrix with randomly positioned delta functions that describe the spatial arrangement of *M* scatterers in a medium, which can be expressed as
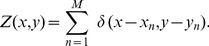
(3)


The phantom was of the size 3 cm
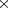
3 cm having a scatterer concentration of 32 scatterers/mm^2^. A 5-MHz Gaussian incident pulse in the *y* direction with a pulse length of 0.45 mm and a beam width of 0.9 mm was produced using
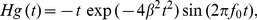
(4)where *f*_0_ is the center frequency and *β* is the bandwidth. The beam width along the *x* direction was determined according to the far-field power density, as provided in [14] and expressed as
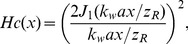
(5)where 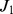 denotes a first order Bessel function, *a* and 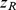 respectively represent the radius and focal length of the transducer, and 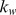 is the wave number. Consequently, simulated ultrasonic RF signals were obtained according to the convolution of an incident wave with the computer phantom, and then demodulated using the Hilbert transform.

It is known that the backscatter statistics of a fully developed speckle follow the Rayleigh distribution corresponding to a speckle SNR of 1.91. In a previous study [9], we demonstrated that the simulation settings used in the current study can produce the envelope statistics of the Rayleigh distribution and an SNR value of 1.91. The histogram (described using bins  = 40) of the envelope data was used for estimating Shannon entropy by using
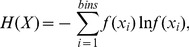
(6)where 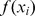 is the PDF of the *i*th bin. A total of 1000 simulation tests were performed to compare the relationships between the SNR and the entropy. The results revealed that the entropy equals 4.8 when the backscatter statistics form a Rayleigh distribution corresponding to an SNR of 1.91, meaning that the property of the backscatter data is random. Because an increase in the entropy means a change in the signal properties from regular, random, to complex, it can be inferred that entropy <4.8 represents a partially developed speckle (regular), and that entropy >4.8 indicates a fully developed speckle with clustered scatterers (complex).

### Acquisition of Backscatter Data from a Lens

In this study, we used the experimental raw image data acquired in our previous study [7] to validate the proposed concept. A brief introduction to sample preparation and the procedure of backscatter data acquisition is described as follows. Five lenses from porcine eyes collected from a local slaughterhouse were used. To induce the cataract, the lenses were immersed in a solution of ethanol, 2-propanol, and formalin at a ratio of 3:3:4 [15]. The hardness of the cataract lens was evaluated by measuring the Young's modulus and using a commercial mechanical device (ElectroForce 3100 Test Instrument, Bose Corporation, Minnesota). The Young's modulus for each specimen at the immersion times of 40, 80, 120, and 160 min was computed based on the slope of the stress-strain curve.

**Figure 2:**
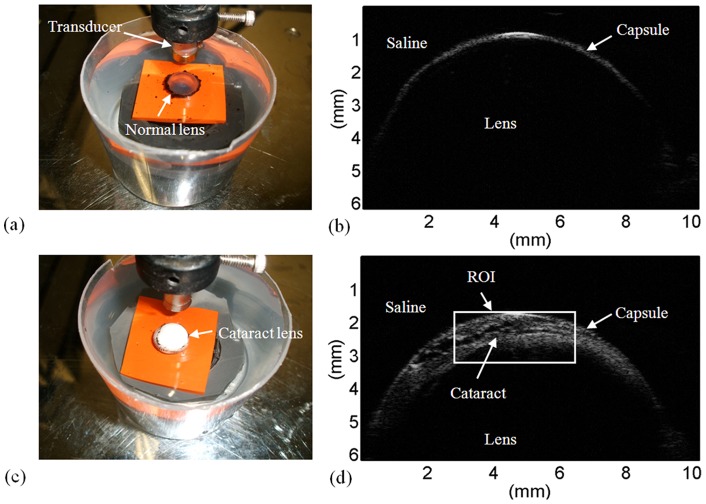
A normal and a cataract lens are shown in (a) and (c), respectively. The B-mode images corresponding to (a) and (c) are shown in (b) and (d), respectively.

An ultrasonic transducer (NIH Ultrasonic Transducer Resource Center, USC, CA) was used at a central frequency of 35 MHz. Both the transducer and lens samples were immersed in a saline buffer solution at a room temperature of approximately 25°C. The transducer movement was controlled using a motor stage and controller (Parker Hannifin Corp., Cleveland, OH) to scan the lens samples. An ultrasonic pulser (AVB2-TB-C, Avtech Electrosystems Ltd., Ottawa, Ontario, Canada) was used to drive the transducer. The image RF signals received from the lens were subsequently amplified using a low-noise amplifier (Miteq 1166, Miteq Inc., Hauppauge, NY), filtered using a bandpass filter (Model BIF-50, Mini-Circuits, Brooklyn, NY), and digitalized using an analog-to-digital converter (CS14200, Gage Applied Technologies, Inc., Lachine, Quebec, Canada) at a sampling rate of 200 MHz. For each lens at the immersion times of 40, 80, 120, and 160 min, the image scanning was performed to acquire 1000 A-lines of backscatter signals from the lens; the distance between each scan-line was 12 µm. Each scan-line was then demodulated to obtain the envelope image of the lens. The B-mode image is displayed based on the log-compressed envelope image with a dynamic range of 40 dB. Figures 2 (a) and (c) show a normal and cataract lens, respectively. The B-mode images of normal and cataract lens are shown in Figures 2 (b) and (d), respectively.

Using the *in vitro* cataract model, cortical cataract was induced along the capsule of a lens. During ultrasound scanning, the focal zone of the transducer was located at the lens capsule so that the major portions of the lens with cataract were scanned. The thickness of the cataract layer generated beneath the capsule was approximately 1 mm, as shown in Figure 2. Therefore, the beam divergence effect of the transducer may not have a significant effect on the ultrasound backscattered signals.

### Entropy Image Formation

**Figure 3:**
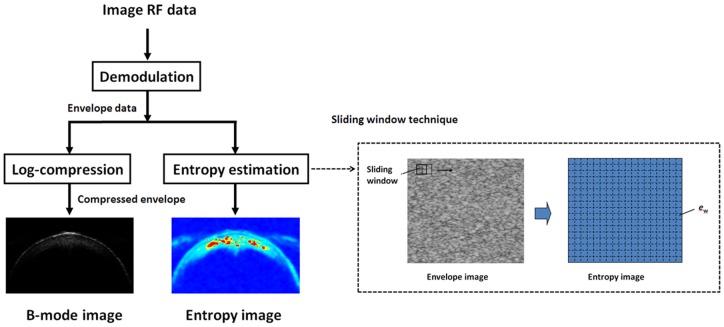
The algorithmic procedure for the construction of ultrasound entropy images.

The entropy image was constructed using a square sliding window to process the envelope image with no log-compression (Figure 3). A square window within the lens envelope image was used to collect the local backscatter envelopes for estimating the local entropy *e*_w_. The parameter *e*_w_ was assigned as the new pixel located in the center of the window. The window was moved throughout the entire envelope image in steps of one pixel, and the above step was repeated. The entropy image of the cataract lens was subsequently constructed using the map of the parameter *e*_w_. Based on the recommendations of a previous study [9], the size of the sliding window was set as the square with a sidelength corresponding to three times the transducer pulse length (i.e., 231 µm) to collect enough numbers of envelope data points for a stable parameter estimation. In the entropy image, the parameters *e*_w_ <4.8 were assigned blue shades varying from deep to shallow with an increasing parameter value, representing various regular characteristics of the scatterers. When *e*_w_  = 4.8, the shade was yellow to express the random property of the scatterers, and those higher than 4.8 were assigned the shades of progressive red in accordance with the increasing parameter value, indicating the incremental degree of clustering in the scatterers.

## Results

**Figure 4:**
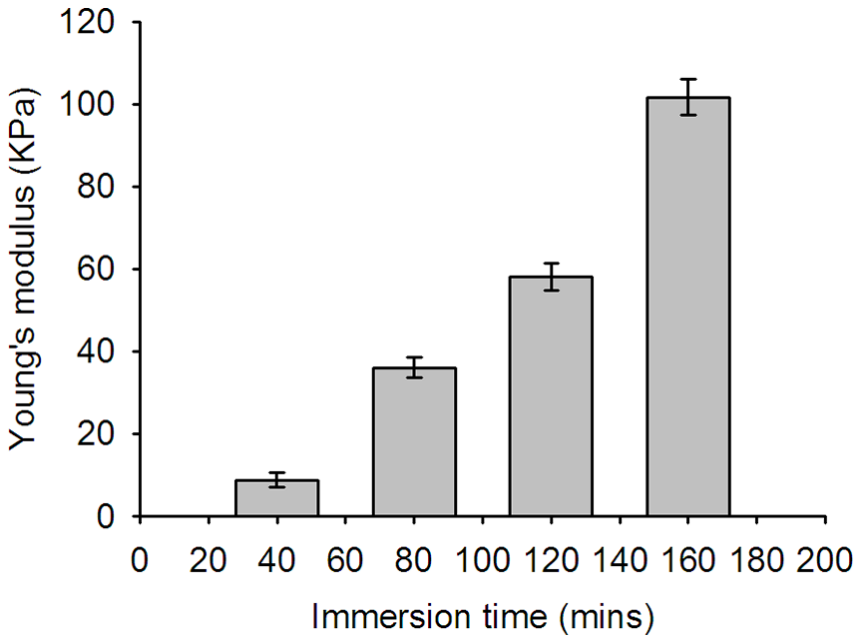
Average Young's modulus as a function of the immersion time. This result indicates a successful induction of cataract lens.

**Figure 5:**
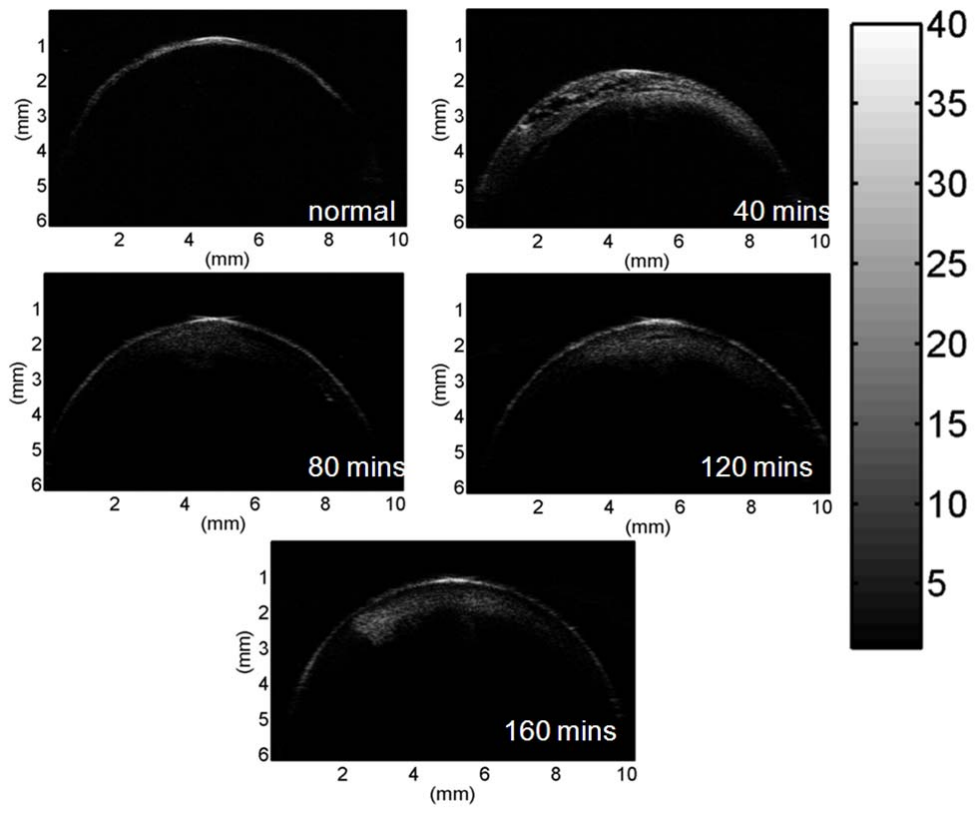
Representative B-mode images of the porcine lens at different stages of cataract formation.

**Figure 6:**
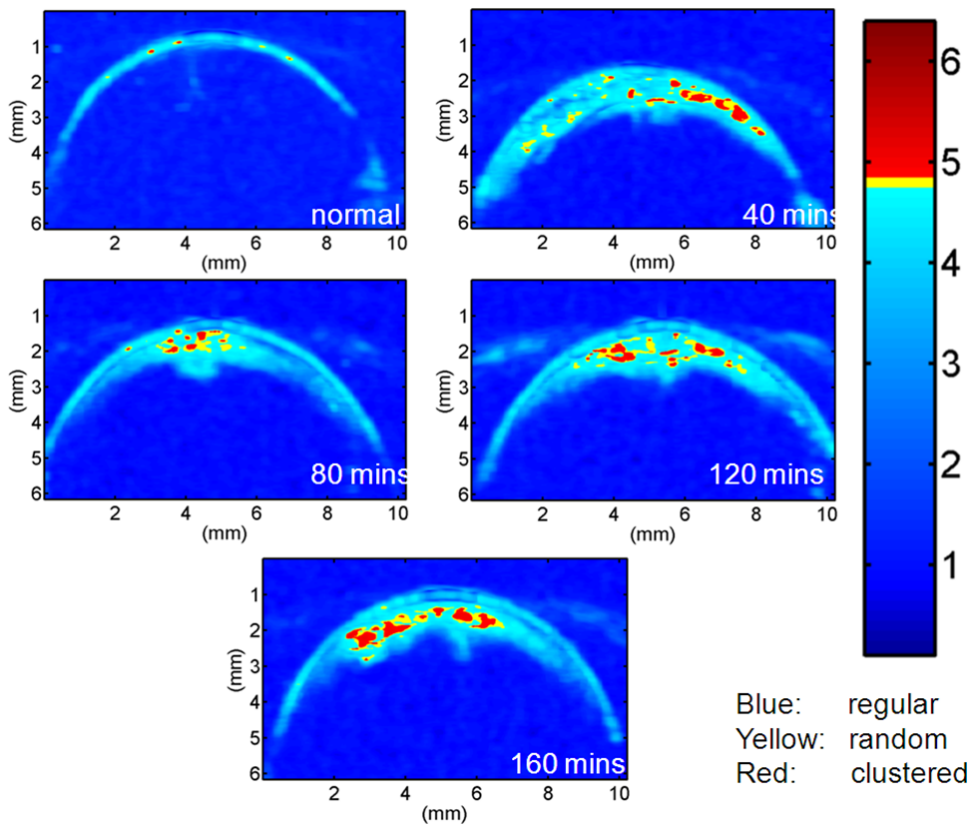
Representative entropy images of the porcine lens at different stages of cataract formation.

Figure 4 shows the results of the lens stiffness during cataract induction. The Young's modulus of the lens increased from approximately 8 to 100 kPa as the immersion time was increased from 40 to 160 min (correlation coefficient *r* = 0.99), indicating that cataract induction increases the stiffness of the lens. Figure 5 shows the B-mode images of the lens for different immersion times. Figure 6 shows the entropy images corresponding to the B-mode images. Most of the deep blue shadings in the entropy images corresponded to the background (saline buffer solution) and the transparent portions of the lens. The light blue shading was more evident in the cataract regions in the entropy images for shorter immersion times, and increasing the immersion time resulted in more red shading in the ROIs of the entropy images. This indicated that the entropy image has the ability to describe different stages of lens cataracts.

**Figure 7:**
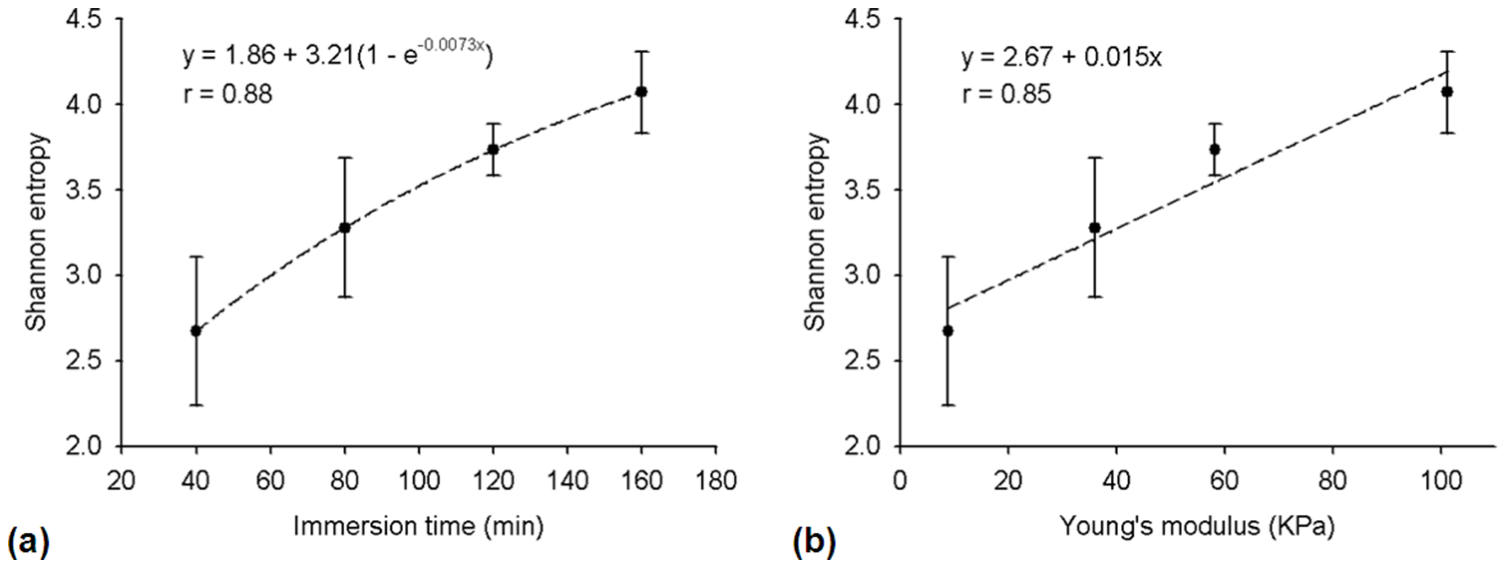
Average entropy as a function of immersion time is shown in (a), and average local entropy as a function of Young's modulus is shown in (b).

To confirm the experimental results, we calculated the mean value of *e*_w_ in the ROI, 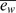 as a function of immersion time (Figure 7 (a)). The value of 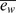 increased from approximately 2.7 to 4.0 as the immersion time was increased from 40 to 160 min (*r* = 0.88). This indicated that the speckle patterns varied from a partially developed speckle to a fully developed speckle, and that the scatterer properties changed from regular to random as the immersion time was increased. Figure 7 (b) shows that 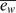 increased from 2.7 to 4.0 as the Young's modulus increased from 8 to 100 kPa (*r* = 0.85). The high correlation coefficient shows that the entropy image is suitable for locally distinguishing variations in the hardness of a cataract lens. Moreover, the overall lens hardness can be determined by calculating the mean of the 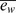 values in the ROI in an entropy image.

## Discussion

### Change in entropy during cataract formation

The protein aggregation and fiber coemption that occur in a lens in the presence of a cataract increase the lens hardness. The current results demonstrate that ultrasound entropy imaging is promising for identification of lens stiffness during cataract induction. Because an entropy image is constructed using the envelope of raw backscatter signals, the mechanism that enables an entropy image to detect the variation of lens hardness can be explained based on the interaction between an ultrasound wave and the scatterers in the scattering medium.

In general, a biological tissue can be modeled as a collection of scatterers. Cataract formation involves changing the arrangements and concentrations of the scatterers (i.e., protein and fibers). Therefore, detecting the variation in the scatterer structures of a lens is integral to determining the hardness of a cataract lens (Figures 6 and 7). For a normal lens, the entropy of backscatter envelope is very small. The global and local entropies of the backscatter envelope in the early stage of cataract formation (i.e., with a short immersion time) have significant regular characteristics (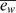 <3.0 in the ROI, with *e*_w_ <3.0 mostly). This is because the scatterer concentration is comparatively low in the cataract region of the lens, which corresponds to minor protein aggregation and fiber coemption being present at the initial stage of cataract formation. For cataracts at later stages, the global entropy of the backscatter envelope of the ROI gradually approaches a Rayleigh distribution (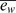 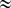
4.8 in the ROI), with the local entropy of the backscatter envelope represented by different degrees of clustered characteristics (*e*_w_ >4.8 mostly). Evidently, the long immersion time required to form a cataract lens induces more pronounced protein aggregation and fiber coemption, and reduces the scatterer spacing, generating a clustered structure of the scatterers in the lens.

### Comparison between entropy and model-based statistical parametric images

We compared the entropy image and ultrasound statistical parametric image based on the Nakagami distribution model that was previously proposed to detect lens cataract [7]. According to the previous study and the current findings, both the entropy image and Nakagami statistical parametric image can reflect different degrees of a lens cataract. However, they have different physical meanings. Ultrasound Nakagami imaging is constructed using the Nakagami parameter map. The Nakagami parameter is the shape parameter of the Nakagami statistical distribution estimated according to the second and fourth moments of backscatter envelopes, thus providing an estimate of backscatter statistics for characterizing tissues. Using an ultrasound Nakagami image to visualize backscatter statistics has been demonstrated to characterize tissue effectively in several medical applications [16]–[19]. Compared with the Nakagami image, the entropy image is constructed using the backscatter envelopes to estimate the entropy value, which is a measure of signal complexity and uncertainty. The entropy itself is not a model-based parameter, although it increases as the density of scatterers in the resolution cell increases [11]. In other words, the entropy may reflect the physical meaning of the backscatter statistics to a degree. In our simulation tests, we determined that the entropy threshold corresponding to the backscatter statistics of the Rayleigh distribution is 4.8, thereby enabling the classification of regular, random, and complex properties of the backscatter data. More importantly, the prerequisite for using the Nakagami distribution or other statistical models in scatterer characterization is that data must follow the used distribution. The superiority of the entropy over the statistical parameters is that the entropy can be estimated using any type of data (e.g., backscatter data after log-compression or nonlinear processing), to provide a more flexible operation in practical applications.

### Subresolvable effect

Refer to the previous study again [7]. The subresolvable effect at an interface or a tissue boundary is an unavoidable issue when using the sliding window algorithm to construct an ultrasound statistical parametric image. When the sliding window moves onto the capsule, the window not only covers the signals from the front interface but also contains those from partial background or lens. Because the front interface contributes stronger echoes than the background and lens do, the parameter value of the backscattered envelopes acquired by the window would tend to be smaller, making a window-shaped artifact occur in the parametric image. However, compared with the previous study [7], the window-shaped artifact caused by the subresolvable effect in entropy images is less evident, although it still makes the center of the cataract layer have relatively higher entropy values and the peripheral proportions corresponded to lowers ones (Figure 6). Actually, to further reduce the window-shaped artifact and its possible effects on entropy imaging, a smaller window may be used. This is because entropy is not a model-based parameter, and therefore entropy imaging may have a broader flexibility than the conventional statistical parametric imaging does to use a smaller window to construct a high-resolution parametric image. It should be noted that the entropy threshold for classifying regular, random, and complex should be recalibrated when using different window sizes.

### Considerations of the proposed entropy image

In addition to Shannon entropy, many other entropy definitions have been introduced for quantifying the scattering characteristics, such as the Renyi or Tsallis entropy measures. Furthermore, numerous algorithms have been developed to facilitate robust entropy estimation [20]–[23]. Thus far, no appropriate entropy approach has been proposed for constructing parametric imaging techniques to characterize tissues. The definition of the Shannon entropy used in this study is the easiest method to implement in practical applications. However, the major limitation of using the proposed Shannon entropy is that the number of bins affects the value of entropy. According to Equation (6), it can be expected that using different bins to describe the histogram of envelope data will result in different values of Shannon entropy. Additional studies are necessary to explore the effects of bin numbers in future applications when using the Shannon entropy defined in Equation (6). When using a different bin number, the entropy corresponding to SNR  = 1.91 may have a different value. The entropy threshold for identifying the fully developed speckle may be obtained by conducting calibrations when using different numbers of bins to describe the probability distribution of envelope signals.

## Conclusion

In this study, we explored the usefulness of information-theory derived entropic imaging in characterizing cataract lenses in porcine eyes at 35-MHz. The results show that entropy imaging can be used to distinguish both global and local variations in lens hardness. This is because the scatterer concentration of the fiber coemption, which is mainly responsible for the increase in lens hardness during cataract formation, can be quantified using the entropy technique to describe the uncertainty of ultrasound backscatter data. In contrast to the conventional ultrasound parametric imaging based on the statistical models, constructing the entropy image does not require the backscatter data to follow a specific statistical distribution. Entropy imaging may have greater potential than that of the statistical parametric images in evaluating the structure and elastic properties of a lens and providing a more flexible and feasible approach in determining the optimal energy to apply during phacoemulsification.
